# Supplementary material for: An entropy-based metric for assessing the purity of single cell populations
Source: Nat Commun. 2020 Jun 22;11:3155. doi: 10.1038/s41467-020-16904-3 (PMC7308400; doi:10.1038/s41467-020-16904-3)
Supplement: Supplementary file 1 — Supplementary Information [file 41467_2020_16904_MOESM1_ESM.pdf]

Supplementary Information

An entropy-based metric for assessing the purity of single cell populations

Liu et al.

Supplementary Figures --- 2-20
Supplementary Tables --- 21-26
Supplementary References -- 27

Supplementary Figure 1. S-E model performance on NB distributed datasets.

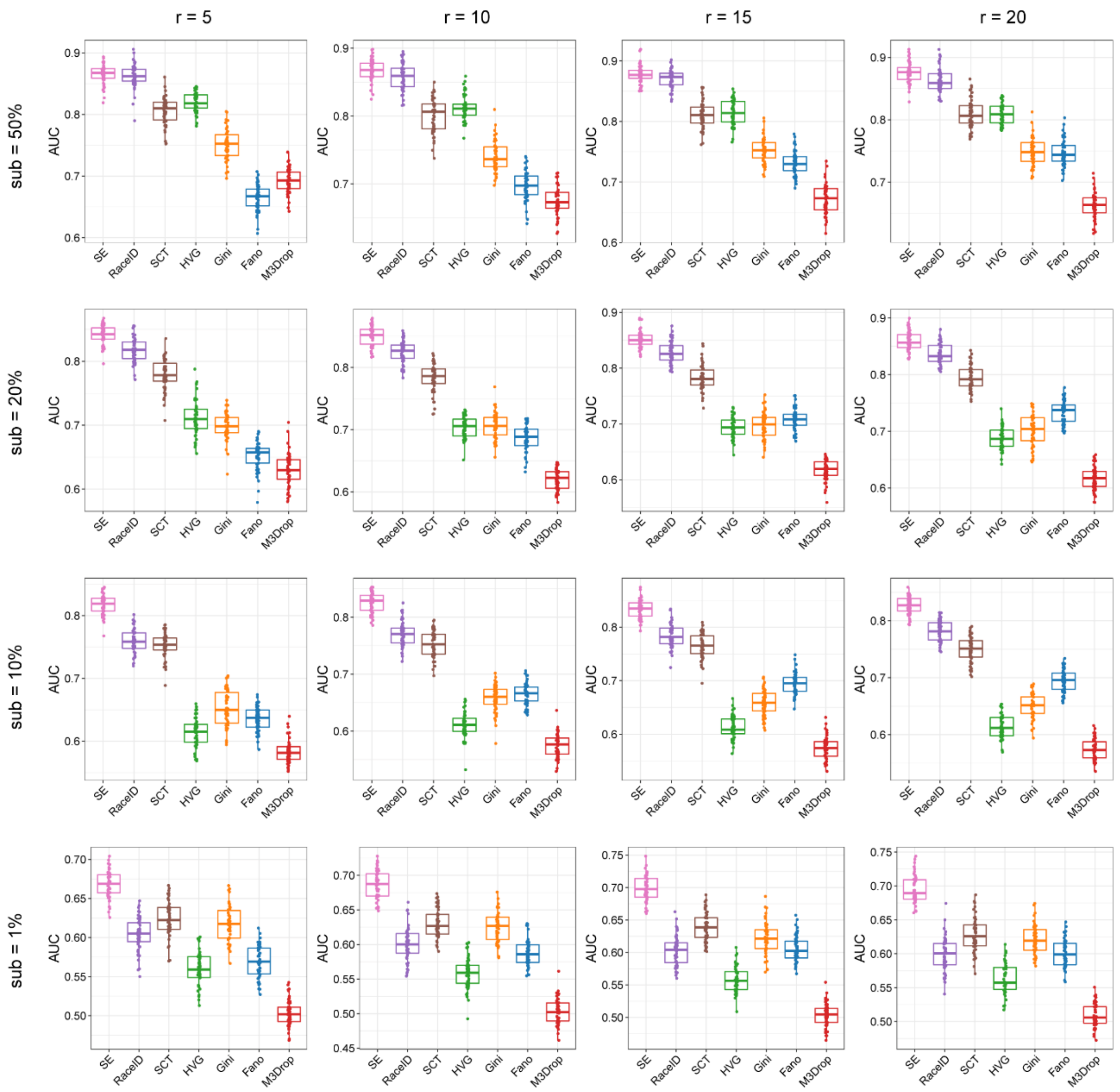

The box plots show the accuracy of S-E model, Gini, HVG and M3Drop in identifying differentially expressed genes on data simulated form NB distribution, corresponding to Fig. 1d (left). The r denotes the dispersion parameter in NB (Methods) and 'sub' represents the fraction of subpopulation. The center line indicates the median AUC value of n=50 repeated runs. The lower and upper hinges represent the 25th and 75th percentiles respectively, and whiskers denote 1.5 times the interquartile range.

Supplementary Figure 2. S-E model performance on ZINB distributed datasets.

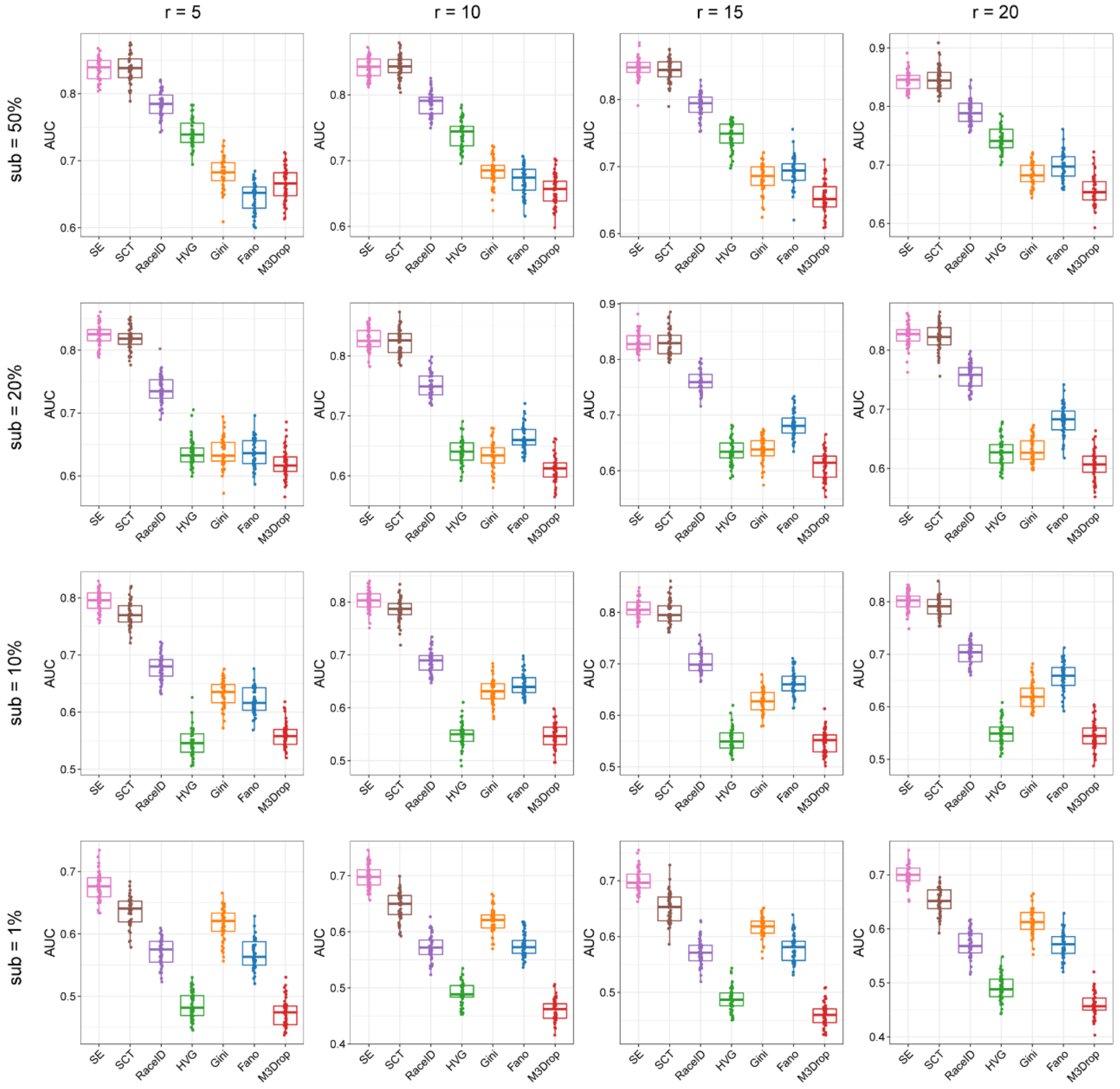

The box plots show the accuracy of S-E model, Gini, HVG and M3Drop in identifying differentially expressed genes on data simulated form ZINB distribution, corresponding to Fig. 1d (right). The center line indicates the median AUC value of n=50 repeated runs. The lower and upper hinges represent the 25th and 75th percentiles respectively, and whiskers denote 1.5 times the interquartile range.

Supplementary Figure 3. Discriminating power of selected genes on droplet based datasets.

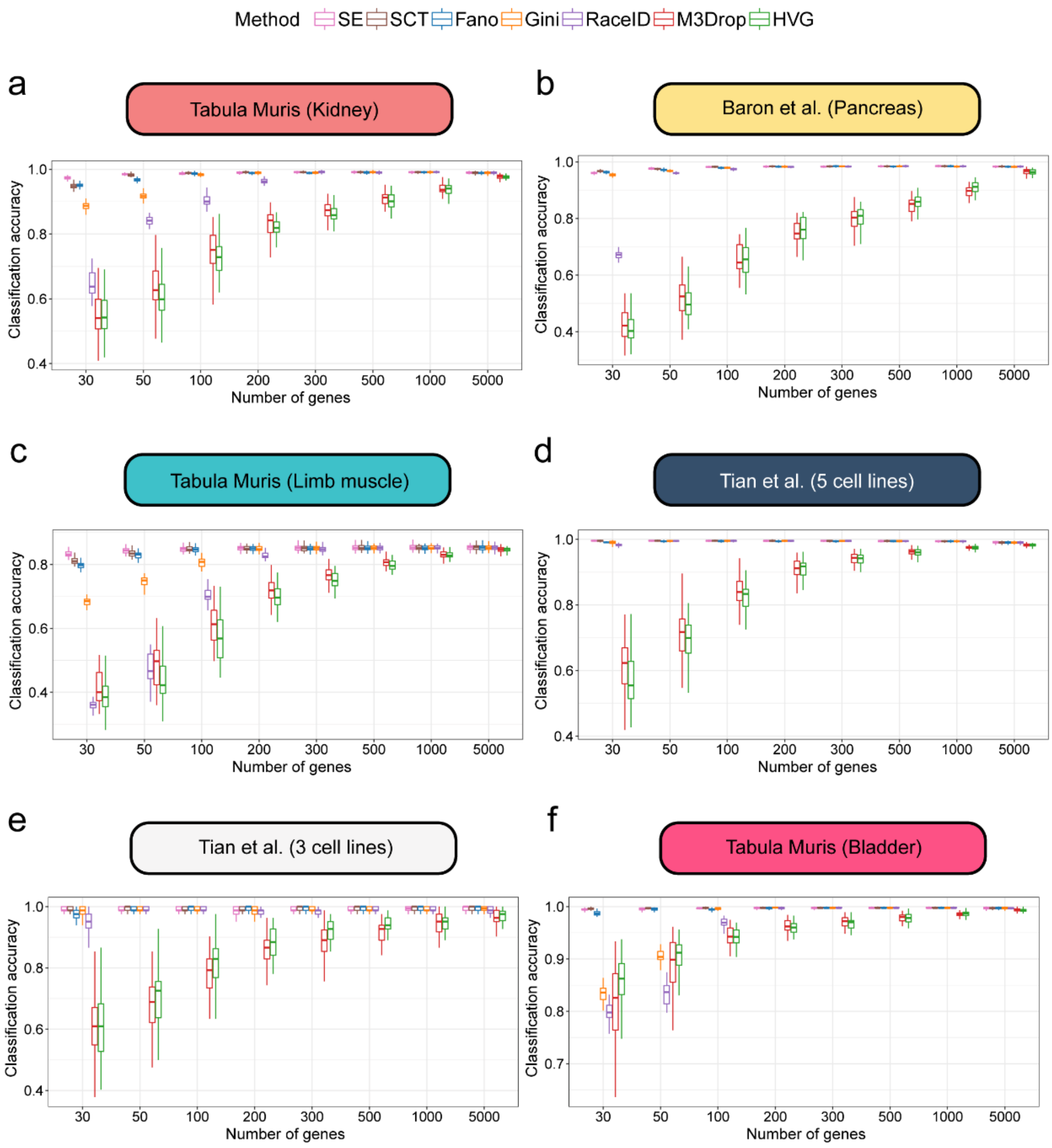

(a-f) The box plots show the classification accuracy of cross-validation tasks using different number of genes selected by S-E model, Gini, HVG and M3Drop, corresponding to Fig. 1e. The cross-validation experiments of these six droplet datasets (Supplementary Table 1) were performed using RF, with 70% cells from the original sample as reference and remaining 30% cells as query set. The center line indicates the median accuracy value of n=50 repeated runs. The lower and upper hinges represent the 25th and 75th percentiles respectively, and whiskers denote 1.5 times the interquartile range.

Supplementary Figure 4. Discriminating power of selected genes on full-length based datasets.

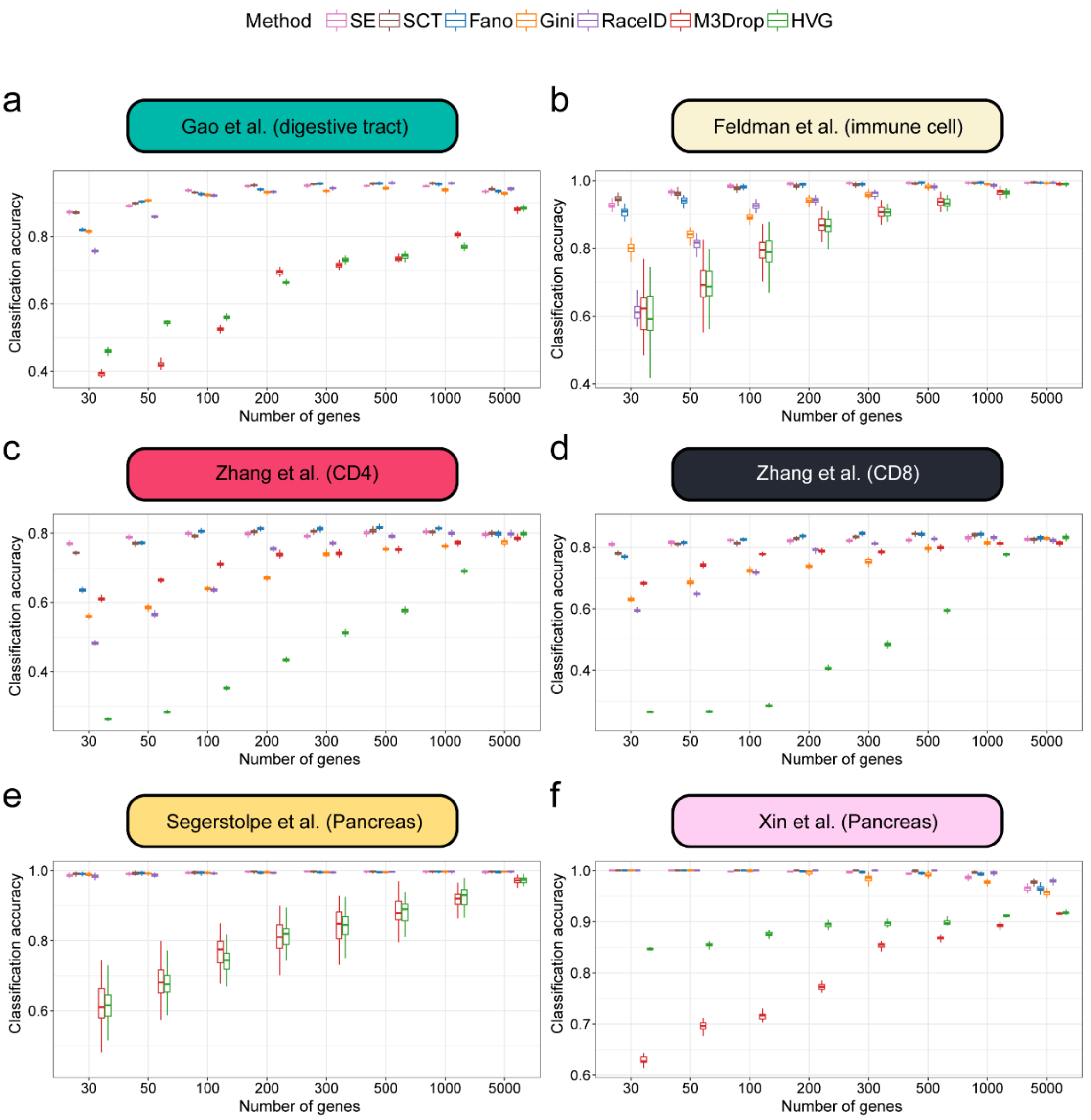

(a-f) The box plots show the classification accuracy of cross-validation tasks using different number of genes selected by S-E model, Gini, HVG and M3Drop, corresponding to Fig. 1f. The center line indicates the median accuracy value of n=50 repeated runs. The lower and upper hinges represent the 25th and 75th percentiles respectively, and whiskers denote 1.5 times the interquartile range. These six datasets shown here were listed in Supplementary Table 1.

Supplementary Figure 5. Reproducibility of features selected by different methods.

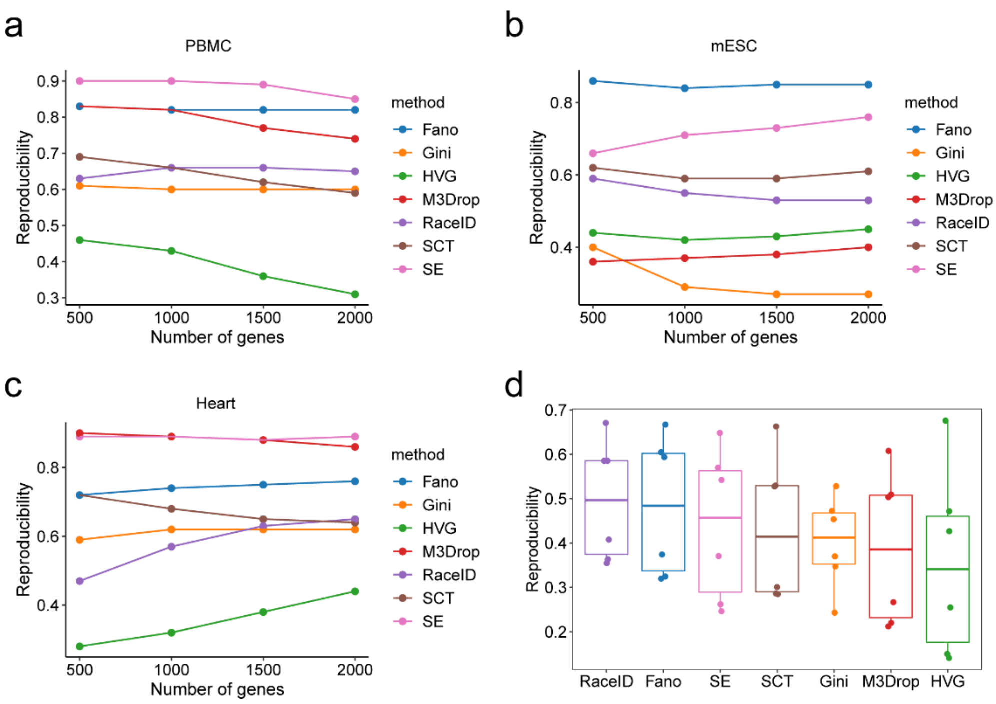

(a-c) Reproducibility of features of **(a)** PBMC, **(b)** mESC, and **(c)** heart replicates (Supplementary Table 3). **(d)** Reproducibility of features across four human pancreas datasets listed in Supplementary Table 3. The center line indicates the median value. The lower and upper hinges represent the 25th and 75th percentiles respectively, and whiskers denote 1.5 times the interquartile range.

Supplementary Figure 6. Datasets used for clustering-based evaluation.

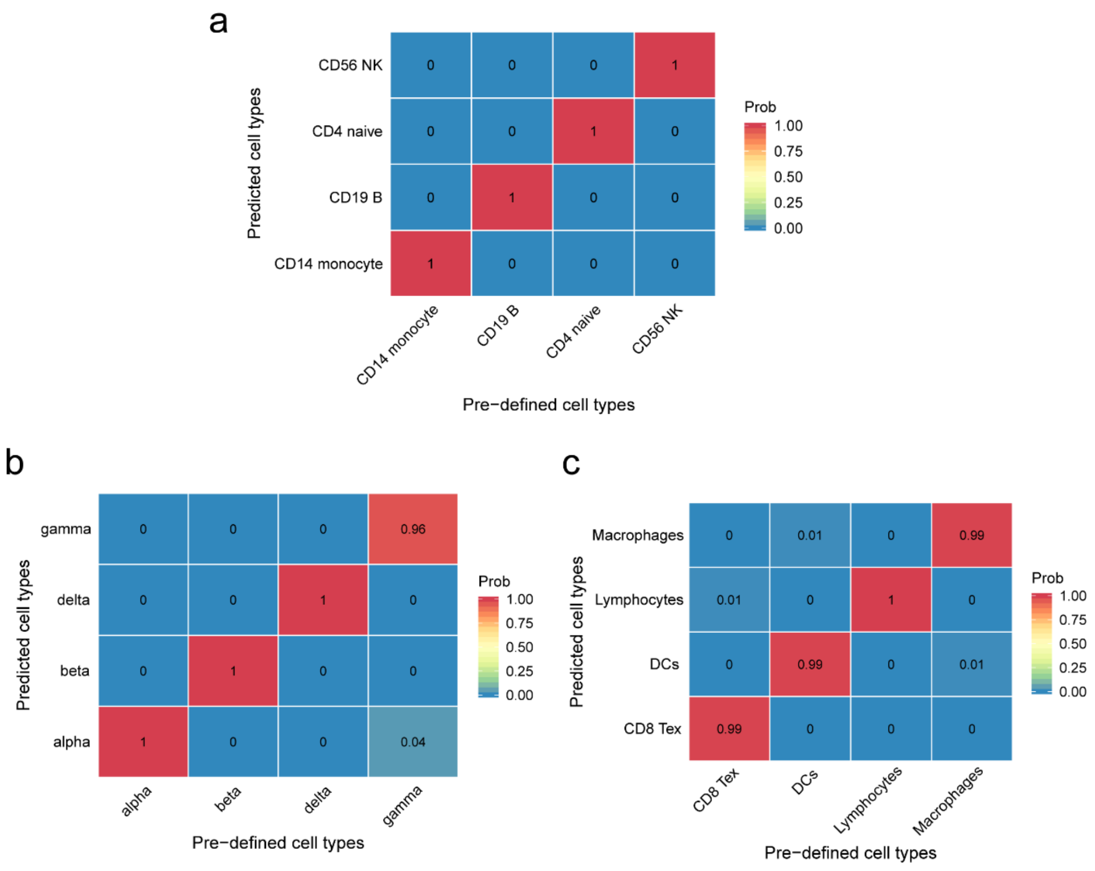

(a,b) Cross-validation experiments of **(a)** the FACS-purified PBMC dataset, **(b)** the pancreas dataset, and **(c)** the immune cell dataset estimated by RF, with 70% cells from the original dataset as reference and the remaining 30% cells as query set. The cell labels were defined with unsupervised clustering by the original publication. We selected 1000 informative genes (based on the reference set) with the supervised feature selection method E-test and repeated the cross-validation procedure for n=50 times for each dataset. The high classification accuracy revealed that these cell types were readily distinguishable.

Supplementary Figure 7. S-E model performance in the context of unsupervised clustering.

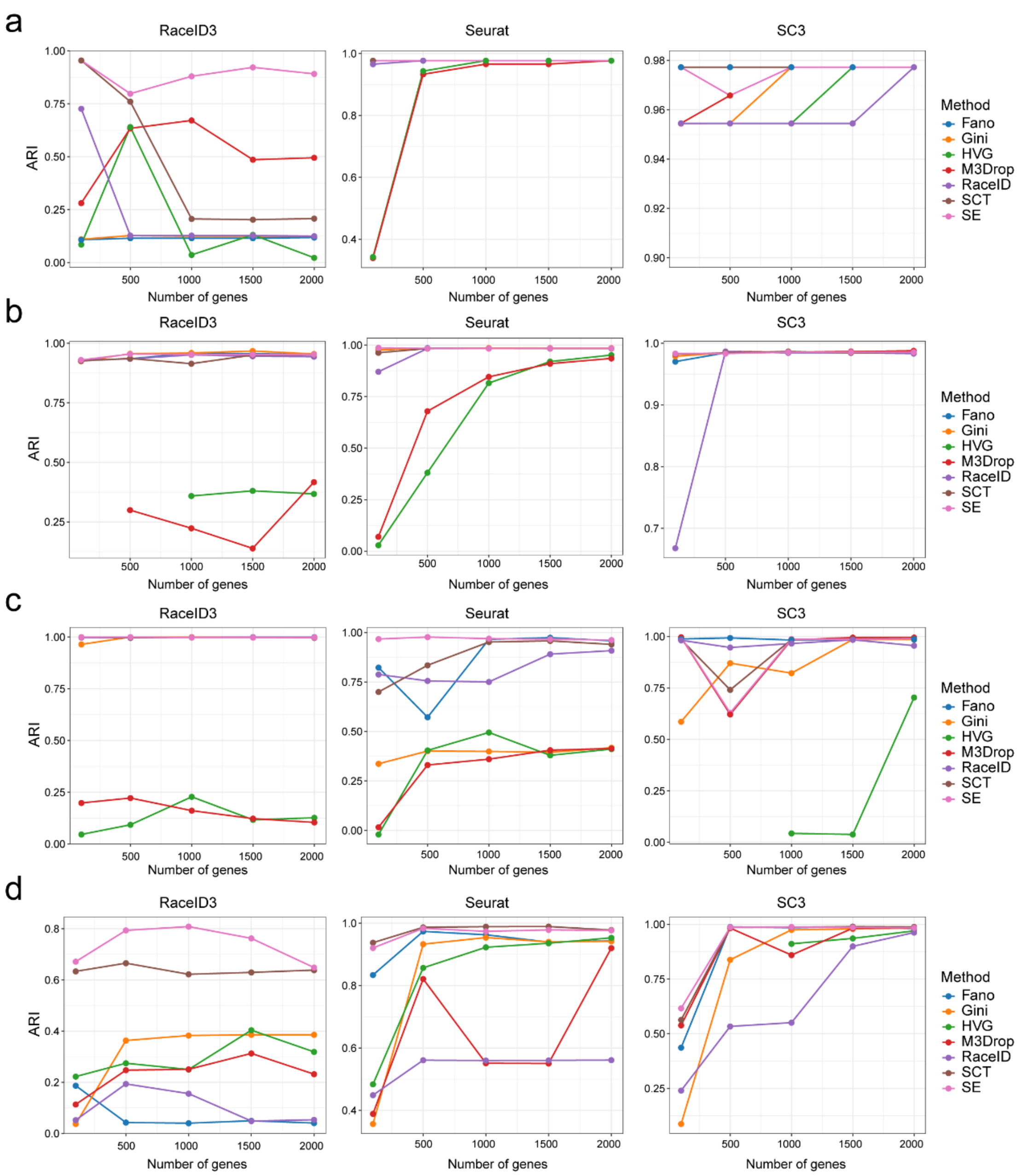

(a-d) ARI for **(a)** 3 cell lines, **(b)** FACS-purified PBMC, **(c)** pancreas, and **(d)** immune cell datasets (Methods) when different feature selection methods were used, corresponding to Fig. 1h.

Supplementary Figure 8. Rare cell type detection based on simulated dataset.

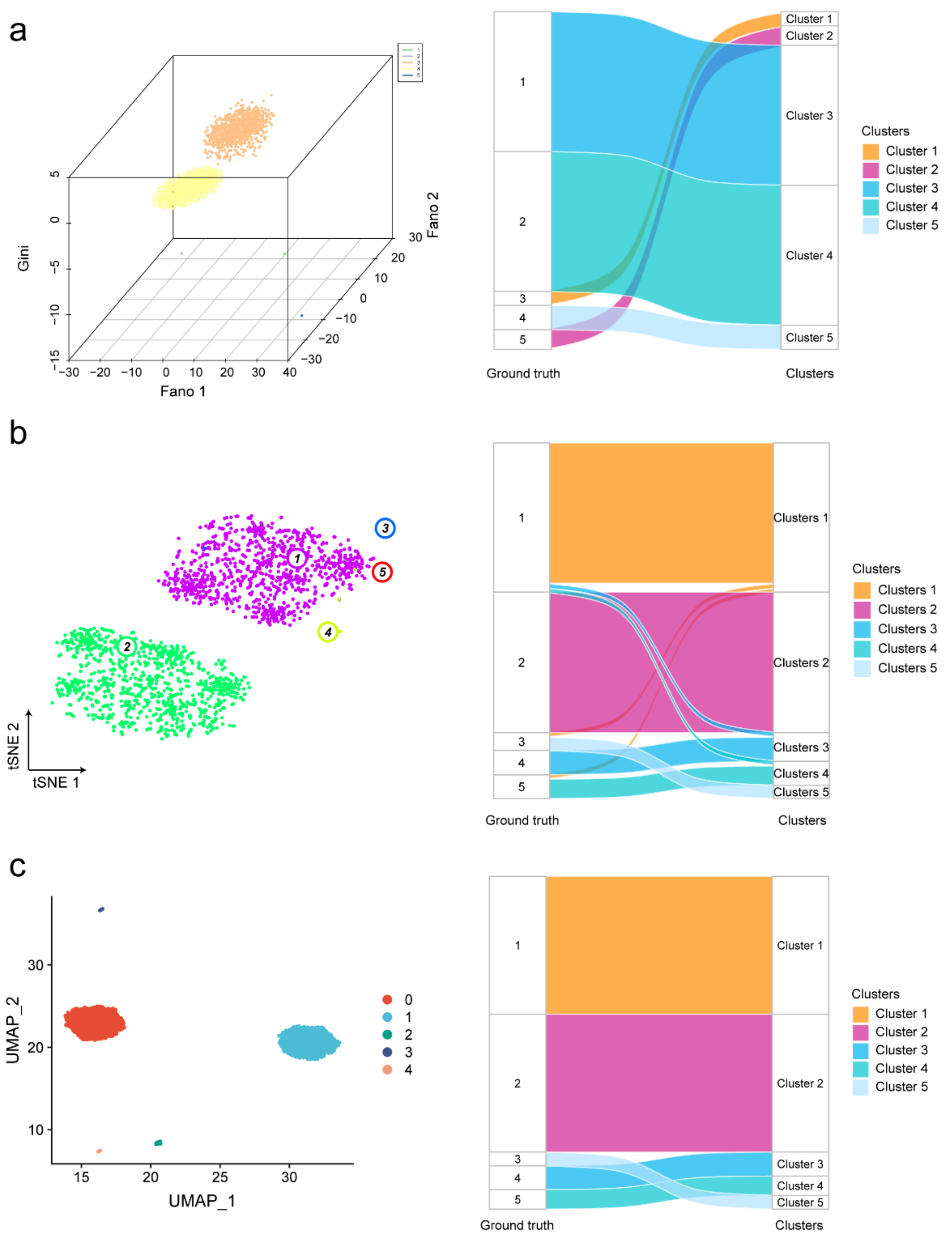

(a) Left, three-dimensional tSNE plot of the GiniClust2 clustering results. Right, Sankey diagram showing the similarity between the true clusters (left-axis) and GiniClust2 clustering results (right-axis). The height of the stratum is proportional to the square root of the number of cells. **(b, c)** Clustering results and performance of RaceID3 **(b)** and S-E model-based Seurat **(c)**.

Supplementary Figure 9. Rare cell type detection based on real dataset.

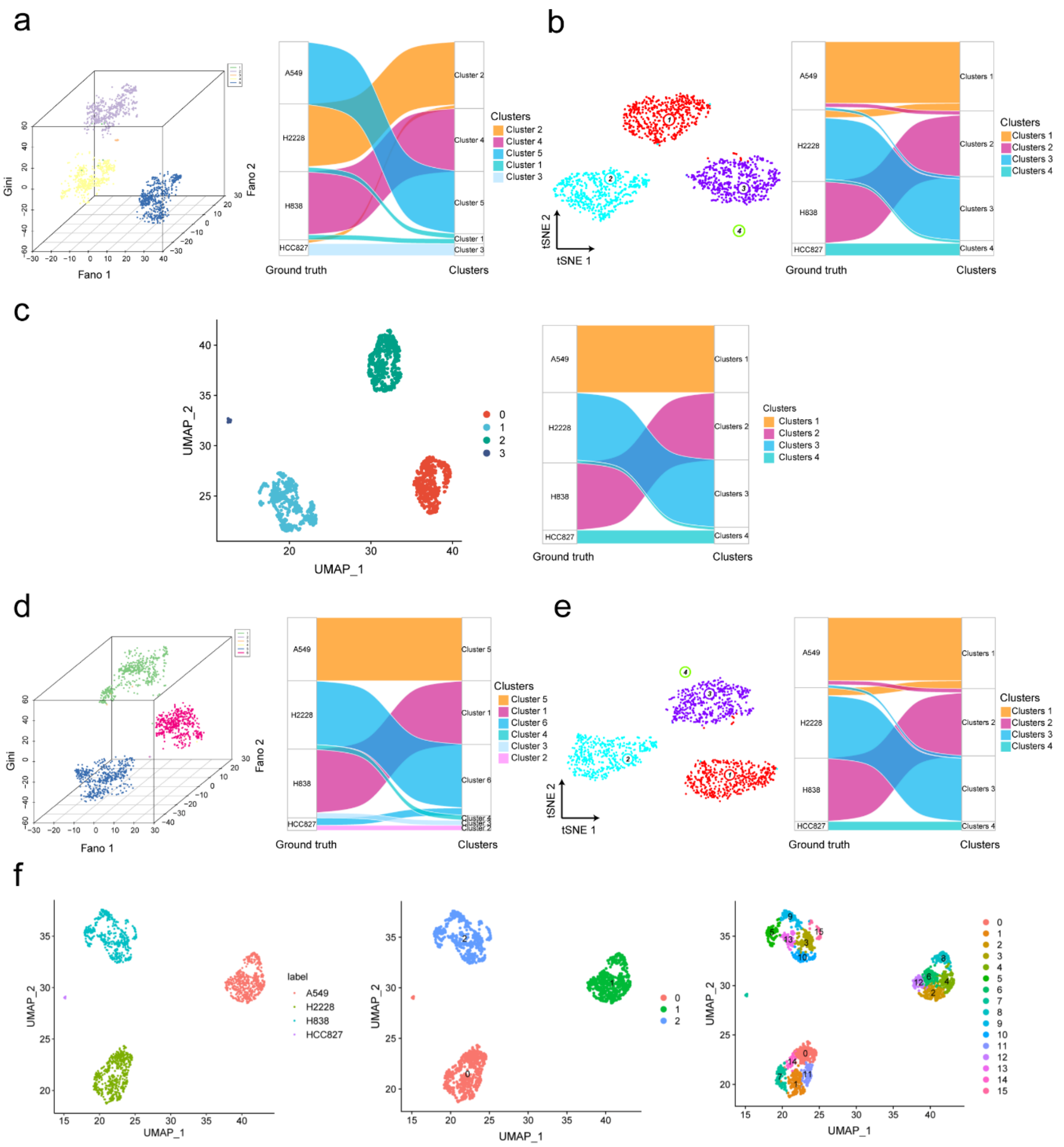

(a) Left, three-dimensional tSNE plot of the GiniClust2 clustering results when there were 20 cells of the rare cell type. Right, Sankey diagram showing the similarity between the true clusters (left-axis) and GiniClust2 clustering results (right-axis). The height of the stratum is proportional to the square root of the number of cells. **(b,c)** Clustering results and performance of RaceID3 **(b)** and S-E model-based Seurat **(c)**, corresponding to **(a)**. **(d,e)** Clustering results when there were 10 cells of the rare cell type of **(d)** GiniClust2 and **(e)** RaceID3. **(f)** UMAP plots of the four cell lines, with color-coded by the true labels (left) and Seurat clustering results (middle and right). S-E model-based Seurat did not effectively identify the rare cell type (n=10).

Supplementary Figure 10. The relationship between ROGUE and the number of informative genes.

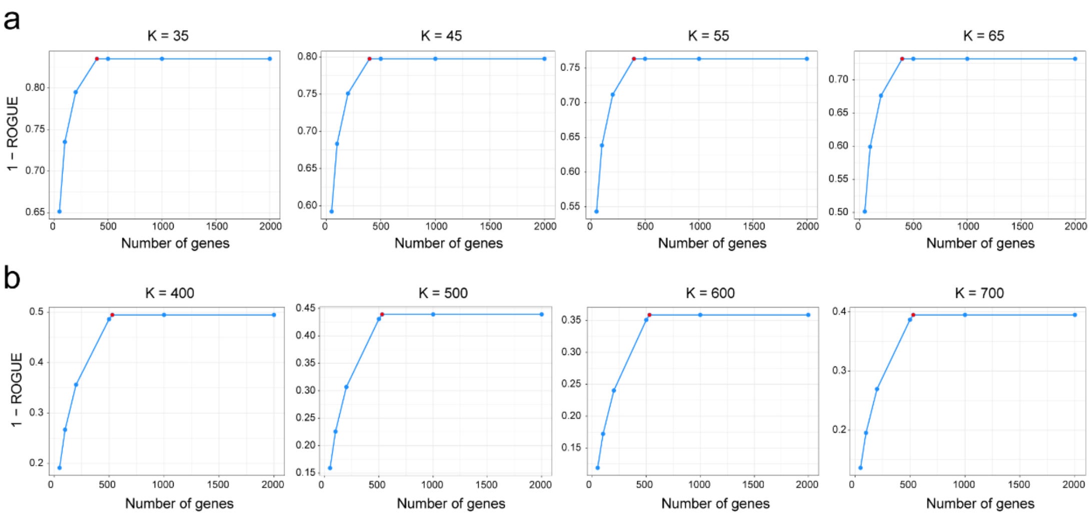

(a,b) Heterogeneity scores (1-ROGUE) versus the number of informative genes for **(a)** the droplet-based dataset and **(b)** the full-length-based dataset. Use of different values of the reference factor K yielded vary similar results.

Supplementary Figure 11. The performance of ROGUE on NB distributed data.

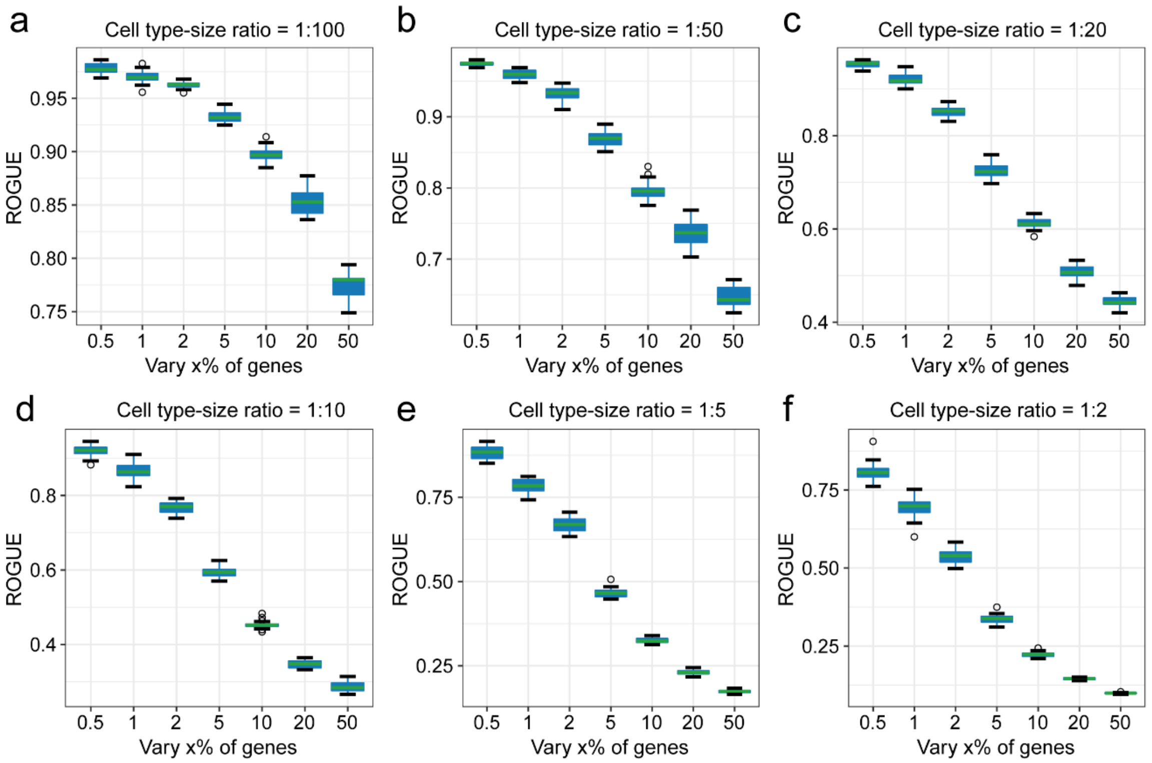

(a-f) The ROGUE index decreases monotonically with increasing varied genes in each simulated mixture consisting of two cell types, with cell type-size ratio ranging from 1:100 to 1:2, corresponding to Fig. 2a. The center line indicates the median ROGUE value of n=50 repeated simulations. The lower and upper hinges represent the 25th and 75th percentiles respectively, and whiskers denote 1.5 times the interquartile range.

Supplementary Figure 12. The performance of ROGUE on ZINB distributed data.

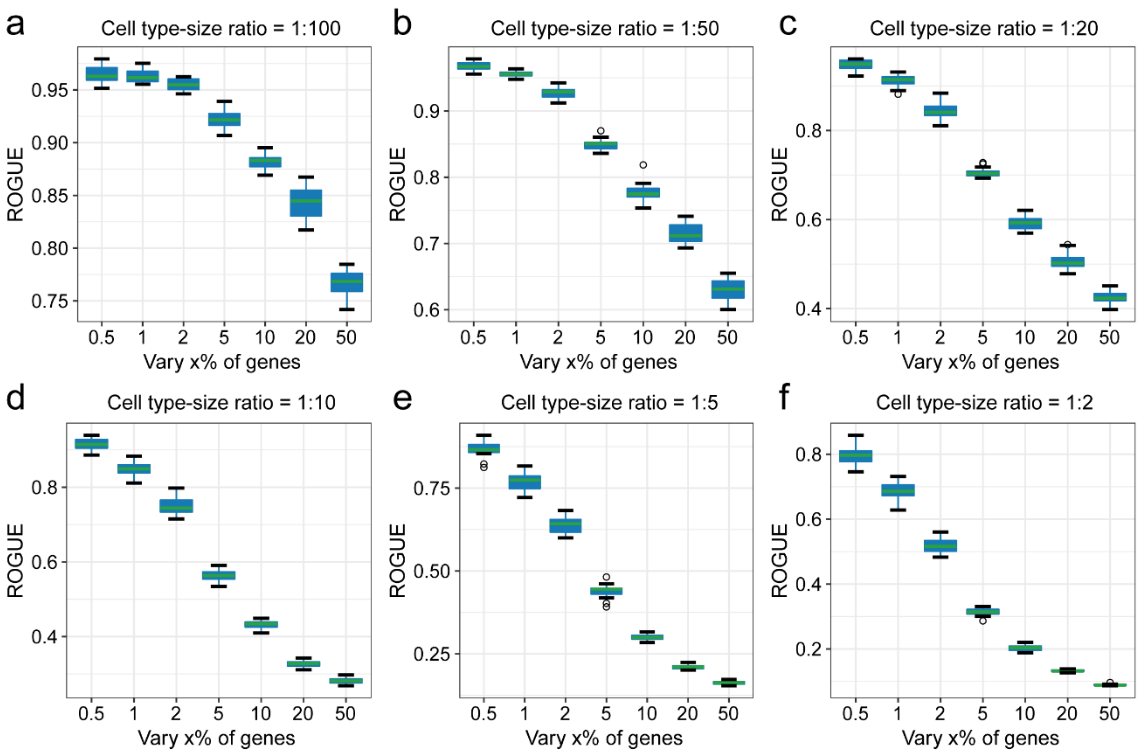

(a-f) The ROGUE index decreases monotonically with increasing varied genes in each simulated mixture consisting of two cell types, with cell type-size ratio ranging from 1:100 to 1:2. The center line indicates the median ROGUE value of n=50 repeated simulations. The lower and upper hinges represent the 25th and 75th percentiles respectively, and whiskers denote 1.5 times the interquartile range.

Supplementary Figure 13. The robustness of ROGUE to the choice of the reference factor K.

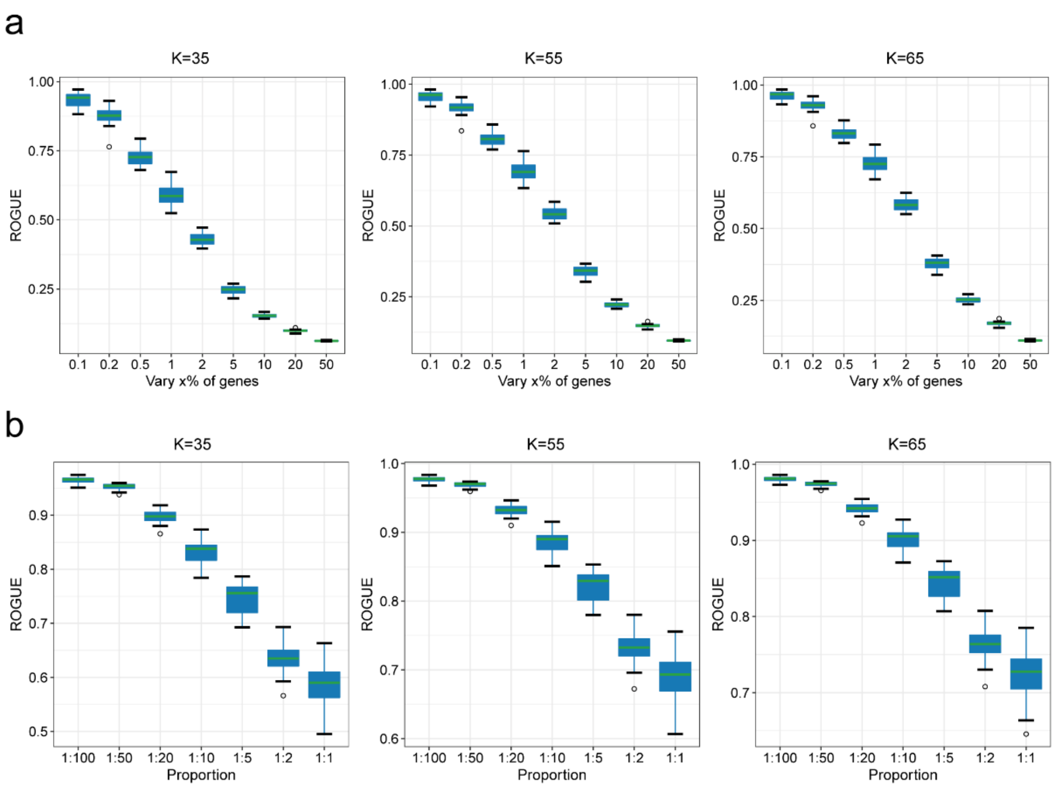

(a) The ROGUE index decreases monotonically with increasing varied genes in each simulated mixture consisting of two cell types (1:1). The center line indicates the median ROGUE value of n=50 repeated simulations. The lower and upper hinges represent the 25th and 75th percentiles respectively, and whiskers denote 1.5 times the interquartile range, corresponding to Fig. 2a. **(b)** The ROGUE values for the simulated mixtures with cell type sizes ranging from 1:100 to 1:1. In each mixture, the number of varied genes was 1% of the total gene number (n = 20,000), corresponding to Fig. 2b.

Supplementary Figure 14. The robustness of ROGUE to sequencing depth and the S-E plot of T cells.

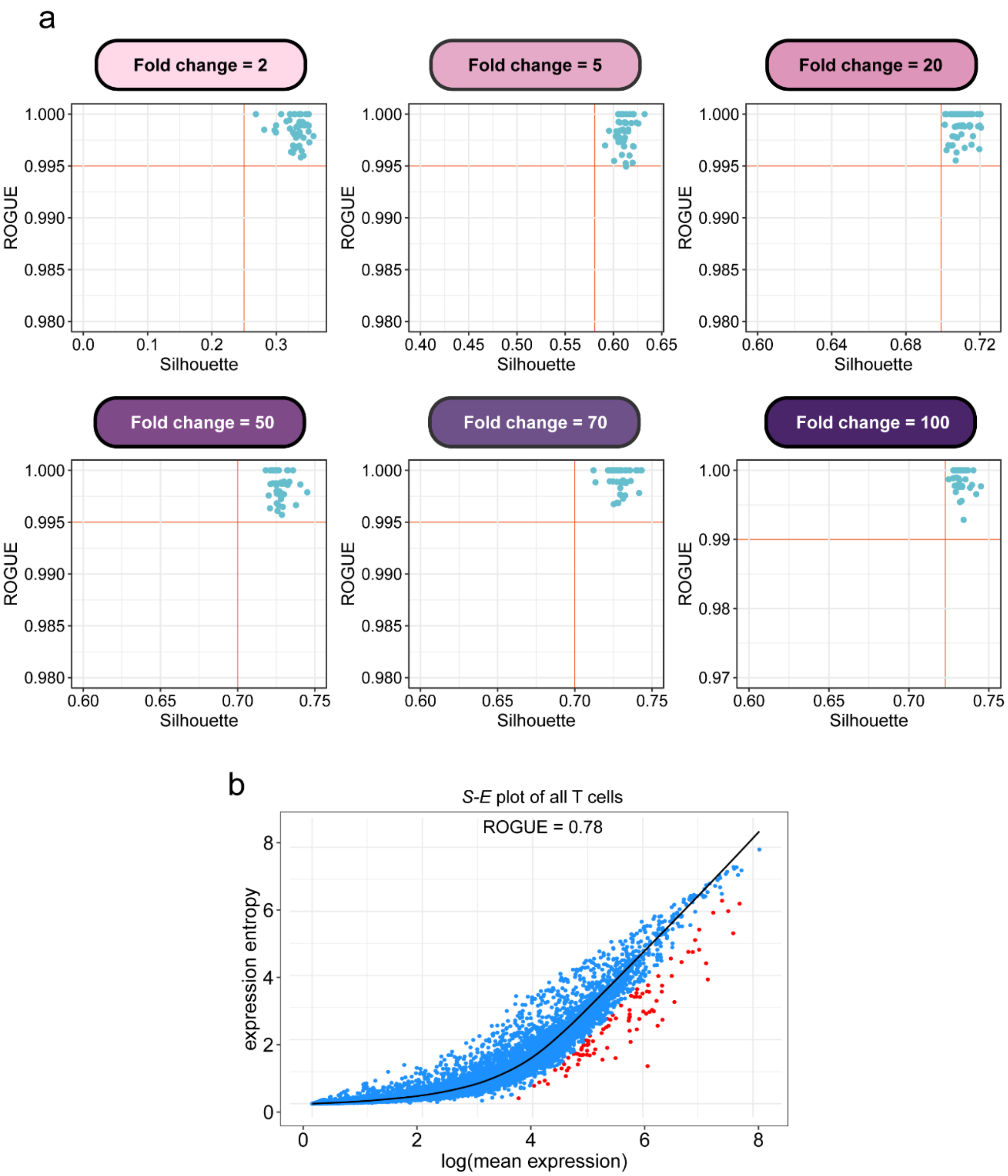

(a) ROGUE values of n=100 mixtures versus the silhouette values for every two replicates within individual mixtures., corresponding to Fig. 2a. Fold change refers to the ratio of the sequencing depth between two replicates. **(b)** Purity assessment of complete human T cells, corresponding to Fig. 2i.

Supplementary Figure 15. ROGUE enhances cell type identification.

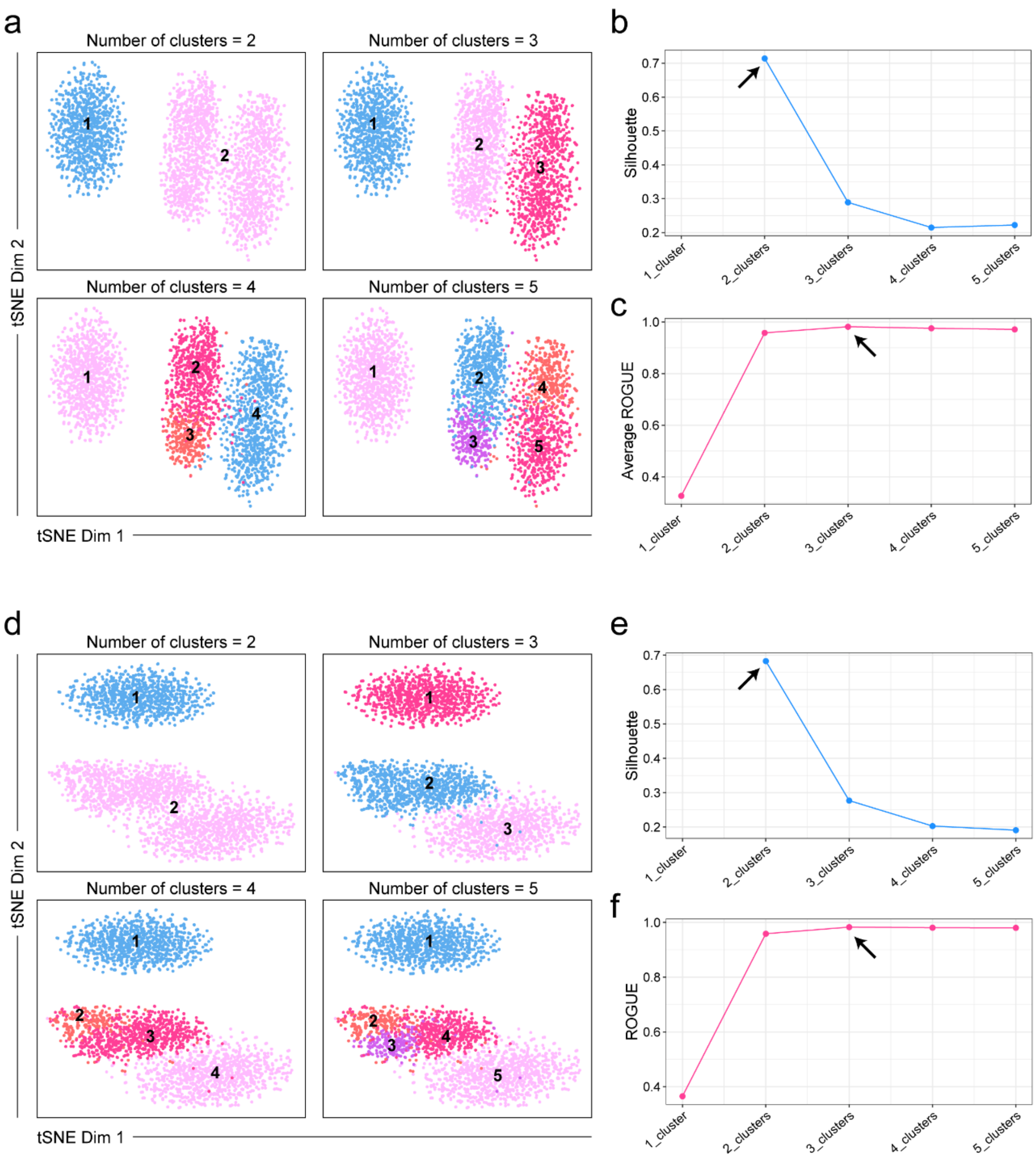

(a) t-SNE plots of a simulated dataset containing three cell types. **(b**,**c)** Corresponding silhouette values **(b)** and average ROGUE values **(c)** when there were 2, 3, 4 and 5 putative clusters respectively. **(d-f)** Another scenario analogous to **(a-c)**, but has varied difference between the three simulated cell types (Methods).

Supplementary Figure 16. ROGUE enhances single cell clustering.

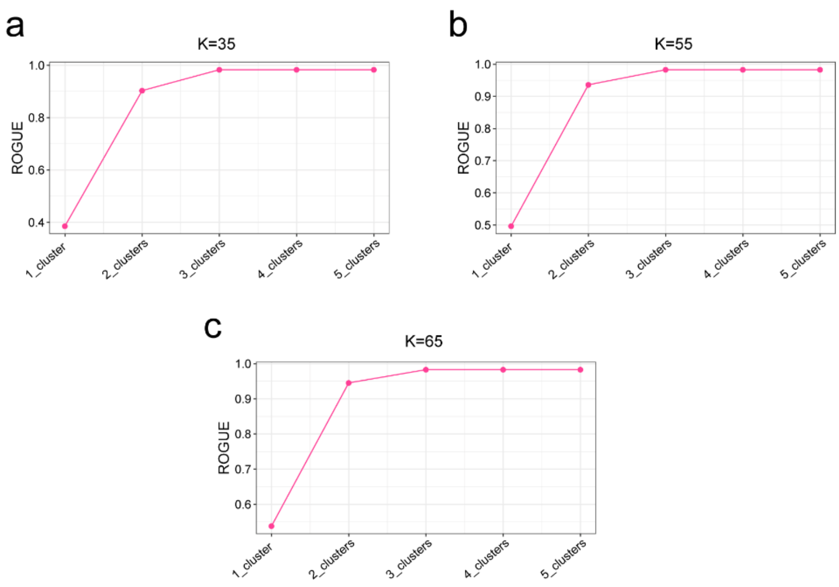

(a-c) Average ROGUE values for the dataset used in Fig.3a when there were 2, 3, 4 and 5 putative clusters respectively, corresponding to Fig. 3c.

Supplementary Figure 17. Cluster information of fibroblasts in original paper and expression levels of signature genes in re-clustered subtypes.

a

Cluster ID	Cluster Name
AF_C0	GABARAP-expressing fibroblasts
AF_C1	lower quality fibroblasts
AF_C2	COL4A2-expressing fibroblasts
AF_C3	normal lung fibroblasts
AF_C4	PLA2G2A-expressing fibroblasts
AF_C5	COL12A1-expressing fibroblasts
AF_C6	TFPI2-expressing fibroblasts

b

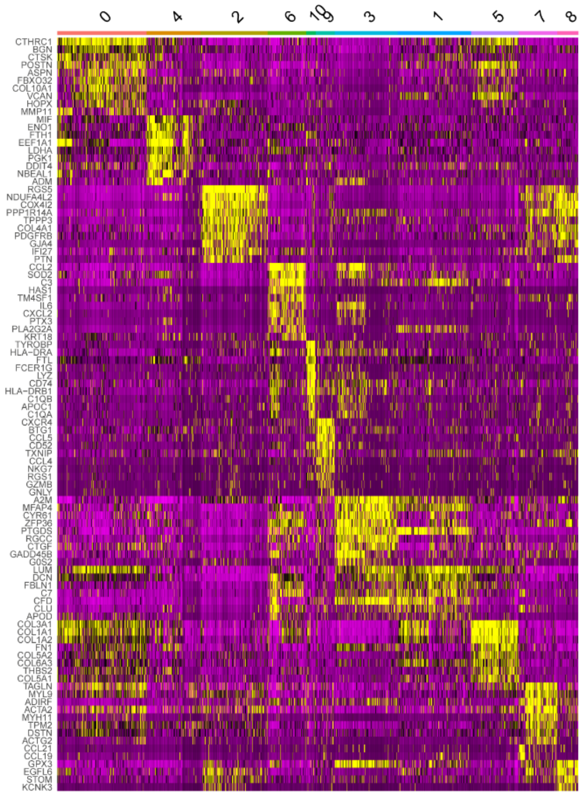

(a) The cluster name of fibroblasts descripted in original paper, corresponding to Fig. 3d (left). **(b)** Gene expression heatmap of re-clustered fibroblast subtypes. Columns denote different clusters and rows denote signature genes.

Supplementary Figure 18. Expression levels of *MKI67* and *STMN1* genes in B cell subtypes.

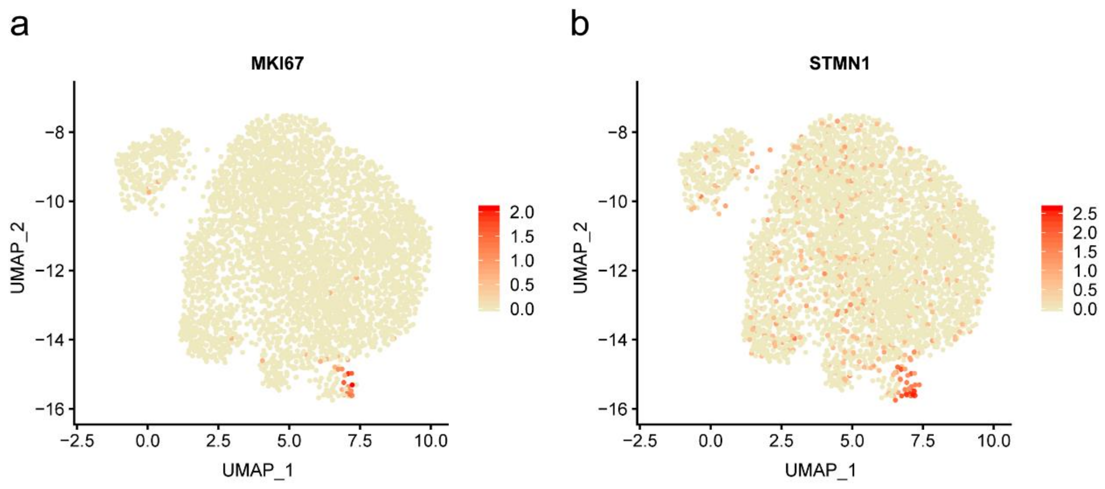

(a,b) t-SNE plots of expression levels of *MKI67* **(a)** and *STMN1* **(b)** in different B cell clusters.

Supplementary Figure 19. S-E plots of Tabula Muris datasets.

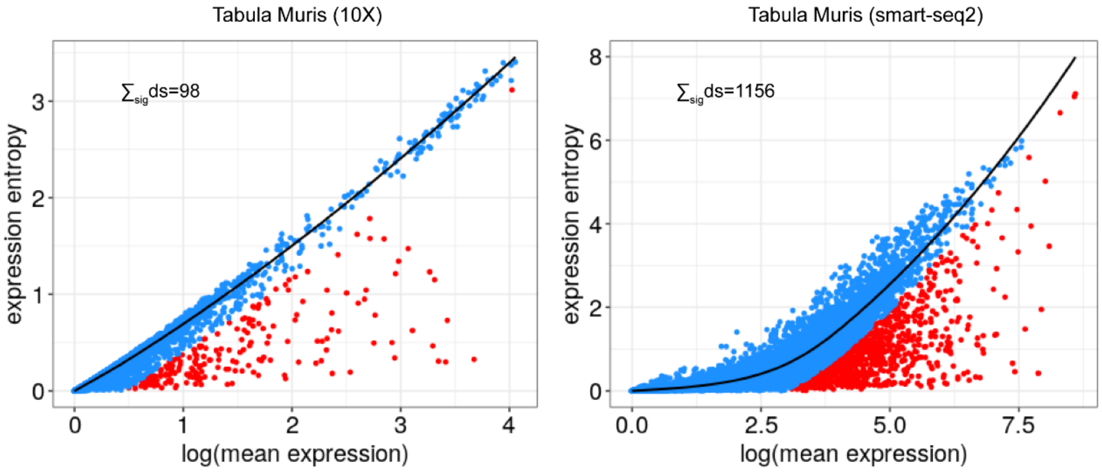

S-E plots of droplet-based dataset (left) and full-length-based dataset (right).

Supplementary Table 1. Datasets used for evaluating the discriminating power of selected genes.

ID	Datasets	GEO accession number	Experimental protocol	Corresponding figure
1	PBMC	SRP073767	10X	Fig. 1e
2	Prefrontal cortex	GSE104276[1]	Smart-seq2	Fig. 1f
3	Tabula Muris (Kidney)	GSE109774	10X	Supplementary Fig. 3a
4	Pancreas	GSE84133[2]	inDrop	Supplementary Fig. 3b
5	Tabula Muris (Limb muscle)	GSE109774	10X	Supplementary Fig. 3c
6	5 cell lines	GSE118767	10X	Supplementary Fig. 3d
7	3 cell lines	GSE118767	CEL-seq2	Supplementary Fig. 3e
8	Tabula Muris (Bladder)	GSE109774	10X	Supplementary Fig. 3f
9	Digestive tract	GSE103239[3]	Smart-seq2	Supplementary Fig. 4a
10	Immune cells	GSE120575	Smart-seq2	Supplementary Fig. 4b
11	CD4 T cells	GSE108989	Smart-seq2	Supplementary Fig. 4c
12	CD8 T cells	GSE108989	Smart-seq2	Supplementary Fig. 4d
13	Pancreas	E-MTAB-5061[4]	Smart-seq2	Supplementary Fig. 4e
14	Pancreas	GSE81608	SMARTer	Supplementary Fig. 4f

Supplementary Table 2. Technical replicates used for reproducibility evaluation.

ID	Tissues	Replicates	Number of cells	GEO/Link
1	mESC	1	2,509	GSE65525[5]
		2	3,443	
2	Brain	1	10K	https://support.10xgenomics.com/single-cell-gene-expression/datasets
		2	1K	
3	Heart	1	10K	
		2	1K	
4	PBMC	1	10K	
		2	1K	

Supplementary Table 3. Four pancreas datasets used for reproducibility evaluation.

ID	Tissues	GEO	Experimental protocol
1	Pancreas	GSE84133	inDrop
2	Pancreas	E-MTAB-5061	Smart-seq2
3	Pancreas	GSE81608	SMARTer
4	Pancreas	GSE85241[6]	CEL-Seq2

Supplementary Table 4. Differences in hallmark pathway activities measured per cell by GSVA, two-sided Student's t-test.

p_val	avg_logFC	p_val_adj	Cluster	Pathway
3.10E-96	4.36E-01	5.18E-94	1	KEGG-RIBOSOME
1.68E-42	1.35E-01	2.80E-40	1	KEGG-CARDIAC-MUSCLE-CONTRACTION
1.20E-30	1.54E-01	2.01E-28	1	KEGG-CALCIUM-SIGNALING-PATHWAY
6.35E-07	1.10E-01	1.06E-04	1	KEGG-GLYCOLYSIS-GLUCONEOGENESIS
7.67E-36	7.12E-01	1.28E-33	5	KEGG-T-CELL-RECEPTOR-SIGNALING-PATHWAY
3.32E-27	1.76E-01	5.55E-25	5	KEGG-NATURAL-KILLER-CELL-MEDIATED-CYTOTOXICITY
1.56E-16	1.62E-01	2.61E-14	5	KEGG-PRIMARY-IMMUNODEFICIENCY
9.05E-10	2.04E-01	1.51E-07	5	KEGG-NON-SMALL-CELL-LUNG-CANCER
7.31E-08	1.70E-01	1.22E-05	5	KEGG-VEGF-SIGNALING-PATHWAY
3.77E-05	1.12E-01	6.30E-03	5	KEGG-FC-EPSILON-RI-SIGNALING-PATHWAY
3.86E-05	1.03E-01	6.45E-03	5	KEGG-ERBB-SIGNALING-PATHWAY
4.44E-05	1.12E-01	7.42E-03	5	KEGG-COLORECTAL-CANCER
9.64E-03	1.11E-01	1.00E+00	5	KEGG-PENTOSE-AND-GLUCURONATE-INTERCONVERSIONS
2.50E-86	2.49E-01	4.18E-84	0	KEGG-ERBB-SIGNALING-PATHWAY
1.78E-79	1.12E-01	2.98E-77	0	KEGG-INTESTINAL-IMMUNE-NETWORK-FOR-IGA-PRODUCTION
4.68E-76	1.49E-01	7.81E-74	0	KEGG-MAPK-SIGNALING-PATHWAY
2.03E-59	2.85E-01	3.40E-57	0	KEGG-COLORECTAL-CANCER
2.85E-08	1.07E-01	4.75E-06	0	KEGG-NON-SMALL-CELL-LUNG-CANCER
4.56E-16	1.68E-01	7.61E-14	2	KEGG-ANTIGEN-PROCESSING-AND-PRESENTATION
3.14E-37	1.54E-01	5.25E-35	3	KEGG-HEMATOPOIETIC-CELL-LINEAGE
2.07E-33	1.83E-01	3.46E-31	3	KEGG-B-CELL-RECEPTOR-SIGNALING-PATHWAY
2.88E-12	1.31E-01	4.81E-10	3	KEGG-ANTIGEN-PROCESSING-AND-PRESENTATION
1.05E-11	1.49E-01	1.75E-09	3	KEGG-MATURITY-ONSET-DIABETES-OF-THE-YOUNG
6.43E-06	1.14E-01	1.07E-03	3	KEGG-LEISHMANIA-INFECTION
2.32E-25	1.59E-01	3.87E-23	4	KEGG-RIG-I-LIKE-RECEPTOR-SIGNALING-PATHWAY
4.65E-18	2.02E-01	7.77E-16	4	KEGG-CYTOSOLIC-DNA-SENSING-PATHWAY
1.96E-04	1.13E-01	3.27E-02	4	KEGG-ANTIGEN-PROCESSING-AND-PRESENTATION
3.56E-15	2.47E-01	5.95E-13	6	KEGG-REGULATION-OF-ACTIN-CYTOSKELETON

1.77E-14	1.70E-01	2.96E-12	6	KEGG-VASCULAR-SMOOTH-MUSCLE-CONTRACTION
5.23E-14	3.24E-01	8.74E-12	6	KEGG-BASE-EXCISION-REPAIR
6.92E-14	3.27E-01	1.16E-11	6	KEGG-LEISHMANIA-INFECTION
4.88E-13	8.28E-01	8.15E-11	6	KEGG-PHENYLALANINE-METABOLISM
2.38E-09	3.28E-01	3.98E-07	6	KEGG-PARKINSONS-DISEASE
2.81E-08	1.87E-01	4.69E-06	6	KEGG-PROTEASOME
3.64E-08	1.63E-01	6.08E-06	6	KEGG-SYSTEMIC-LUPUS-ERYTHEMATOSUS
1.09E-06	2.15E-01	1.81E-04	6	KEGG-PATHOGENIC-ESCHERICHIA-COLI-INFECTION
4.02E-06	4.33E-01	6.71E-04	6	KEGG-LONG-TERM-POTENTIATION
6.63E-06	2.12E-01	1.11E-03	6	KEGG-CYSTEINE-AND-METHIONINE-METABOLISM
7.77E-06	1.76E-01	1.30E-03	6	KEGG-OOCYTE-MEIOSIS
1.23E-05	2.14E-01	2.05E-03	6	KEGG-PENTOSE-PHOSPHATE-PATHWAY
3.29E-05	1.24E-01	5.49E-03	6	KEGG-UBIQUITIN-MEDIATED-PROTEOLYSIS
6.48E-05	1.75E-01	1.08E-02	6	KEGG-B-CELL-RECEPTOR-SIGNALING-PATHWAY
1.11E-04	2.01E-01	1.86E-02	6	KEGG-REGULATION-OF-AUTOPHAGY
1.52E-04	1.63E-01	2.54E-02	6	KEGG-CARDIAC-MUSCLE-CONTRACTION
2.39E-03	1.10E-01	3.99E-01	6	KEGG-ACUTE-MYELOID-LEUKEMIA
6.02E-03	1.39E-01	1.00E+00	6	KEGG-CALCIUM-SIGNALING-PATHWAY
6.68E-03	1.42E-01	1.00E+00	6	KEGG-THYROID-CANCER
9.15E-03	1.02E-01	1.00E+00	6	KEGG-PYRIMIDINE-METABOLISM

Supplementary Table 5. Thirteen signature genes derived from B_C02_ACTB.

p_val	avg_logFC	pct.1	pct.2	p_val_adj	cluster	gene
5.96E-134	0.62	1.00	0.98	6.68E-130	2	ACTB
1.91E-86	0.38	1.00	1.00	2.15E-82	2	TMSB4X
2.33E-67	0.46	0.91	0.74	2.61E-63	2	GAPDH
9.13E-47	0.41	0.90	0.77	1.02E-42	2	ACTG1
1.99E-42	0.35	0.62	0.36	2.23E-38	2	CTSH
8.31E-42	0.37	0.47	0.19	9.32E-38	2	S100A11
1.55E-40	0.46	0.61	0.39	1.74E-36	2	HLA-DRB5
4.51E-40	0.36	1.00	0.99	5.06E-36	2	MT-CO1
6.84E-40	0.50	0.38	0.11	7.67E-36	2	HOPX
1.66E-34	0.51	0.29	0.04	1.86E-30	2	SPP1
1.78E-28	0.30	0.88	0.81	2.00E-24	2	PFN1
1.29E-24	0.27	0.83	0.75	1.45E-20	2	MYL6
3.41E-16	0.32	0.40	0.26	3.82E-12	2	LGALS1

Supplementary References

1. Zhong, S. *et al. A single-cell RNA-seq survey of the developmental landscape of the human prefrontal cortex*. *Nature.* **555**, 524-528 (2018).
2. Baron, M. *et al.* A Single-Cell Transcriptomic Map of the Human and Mouse Pancreas Reveals Inter- and Intra-cell Population Structure. *Cell Syst.* **3**, 346-360.e4 (2016).
3. Gao, S. *et al.* Tracing the temporal-spatial transcriptome landscapes of the human fetal digestive tract using single-cell RNA-sequencing. *Nat. Cell Biol.* **20**, 721–734 (2018).
4. Segerstolpe, Å. *et al.* Single-Cell Transcriptome Profiling of Human Pancreatic Islets in Health and Type 2 Diabetes. *Cell Metab.* **24**, 593–607 (2016).
5. Klein, A. M. *et al.* Droplet Barcoding for Single-Cell Transcriptomics Applied to Embryonic Stem Cells. *Cell* **161**, 1187–1201 (2015).
6. Muraro, M. J. *et al.* A Single-Cell Transcriptome Atlas of the Human Pancreas. *Cell Syst.* **3**, 385-394.e3 (2016).
